# Targeting HIV-1 envelope glycoprotein trimers to B cells using APRIL improves antibody responses

**DOI:** 10.1186/1742-4690-9-S2-P300

**Published:** 2012-09-13

**Authors:** M Melchers, I Bontjer, T Tong, N Chung, P Klasse, D Eggink, M Gentile, A Cerutti, D Montefiori, W Olson, B Berkhout, J Binley, J Moore, R Sanders

**Affiliations:** 1 Weill Medical College of Cornell University, New York, NY, USA

## Background

An HIV-1 vaccine remains elusive, in part because various factors limit the quantity and quality of the antibodies raised against the viral envelope glycoprotein complex (Env). We hypothesized that targeting Env vaccines directly to B cells, by fusing them to molecules that bind and activate these cells, would improve Env-specific antibody responses.

## Methods

We fused trimeric Env gp140 to A PRoliferation-Inducing Ligand (APRIL), B-cell Activating Factor (BAFF), and CD40 Ligand (CD40L).

## Results

The Env-APRIL, Env-BAFF and Env-CD40L gp140 trimers all enhanced the expression of activation-induced cytidine deaminase (AID) expression, the enzyme responsible for inducing somatic hypermutation, antibody affinity maturation and antibody class-switching. They also triggered IgM, IgG and IgA secretion from human B cells in vitro. The Env-APRIL trimers induced higher anti-Env antibody responses in rabbits, including neutralizing antibodies against Tier 1 viruses. The enhanced Env-specific responses were not associated with a general increase in total plasma antibody concentrations, indicating that the effect of APRIL was Env-specific. All the rabbit sera raised against gp140 trimers, irrespective of the presence of CD40L, BAFF or APRIL, recognized trimeric Env efficiently, while sera raised against gp120 monomers did not. The levels of trimer-binding and virus-neutralizing antibodies were strongly correlated, suggesting that gp140 trimers are superior immunogens to gp120 monomers.

## Conclusion

Targeting and activating B cells with a trimeric HIV-1 Env-APRIL fusion protein may improve the induction of humoral immunity against HIV-1. Targeting B cells directly may also be useful for other vaccines.

